# Involvement of Cholinergic, Adrenergic, and Glutamatergic Network Modulation with Cognitive Dysfunction in Alzheimer’s Disease

**DOI:** 10.3390/ijms22052283

**Published:** 2021-02-25

**Authors:** Yu-Jung Cheng, Chieh-Hsin Lin, Hsien-Yuan Lane

**Affiliations:** 1 Department of Physical Therapy and Graduate Institute of Rehabilitation Science, China Medical University, Taichung 40402, Taiwan; chengyu@mail.cmu.edu.tw; 2 Department of Rehabilitation, China Medical University Hospital, Taichung 40402, Taiwan; 3 Institute of Clinical Medical Science, China Medical University, Taichung 40402, Taiwan; 4 Graduate Institute of Biomedical Sciences, China Medical University, Taichung 40402, Taiwan; 5 Kaohsiung Chang Gung Memorial Hospital, Chang Gung University College of Medicine, Kaohsiung 83301, Taiwan; 6 School of Medicine, Chang Gung University, Taoyuan 33302, Taiwan; 7 Department of Psychiatry & Brain Disease Research Center, China Medical University Hospital, Taichung 40402, Taiwan; 8 Department of Psychology, College of Medical and Health Sciences, Asia University, Taichung 41354, Taiwan

**Keywords:** Alzheimer’s disease, cholinergic, adrenergic, glutamatergic, NMDAR

## Abstract

Alzheimer’s disease (AD), the most common cause of dementia, is a progressive neurodegenerative disease. The number of AD cases has been rapidly growing worldwide. Several the related etiological hypotheses include atypical amyloid β (Aβ) deposition, neurofibrillary tangles of tau proteins inside neurons, disturbed neurotransmission, inflammation, and oxidative stress. During AD progression, aberrations in neurotransmission cause cognitive decline—the main symptom of AD. Here, we review the aberrant neurotransmission systems, including cholinergic, adrenergic, and glutamatergic network, and the interactions among these systems as they pertain to AD. We also discuss the key role of *N*-methyl-d-aspartate receptor (NMDAR) dysfunction in AD-associated cognitive impairment. Furthermore, we summarize the results of recent studies indicating that increasing glutamatergic neurotransmission through the alteration of NMDARs shows potential for treating cognitive decline in mild cognitive impairment or early stage AD. Future studies on the long-term efficiency of NMDA-enhancing strategies in the treatment of AD are warranted.

## 1. Introduction

Patients with dementia experience declines in at least two areas of cognition: memory and thinking. Alzheimer’s disease (AD), the most common cause of dementia, is a progressive neurodegenerative disease that causes memory and cognitive impairments as well as behavioral and psychiatric abnormalities [1,2]. AD has gained increasing attention because of the growing burden it places on the global health care system [3]. The development of drugs to treat AD is difficult because the causes of AD remain poorly understood. No new drug or modifying treatment for AD has been approved since 2013 [4].

Patients with AD undergo two main histopathological changes: (i) the extracellular deposition of amyloid plaques, consisting of amyloid β (Aβ), in the brain tissue and (ii) the formation of intraneuronal neurofibrillary tangles of the phosphorylated tau proteins. Although the mechanism through which Aβ and hyperphosphorylated tau damage synaptic dysfunction warrants further clarification, an increasing amount of evidence suggests that the main cause is alterations in the neurotransmitter systems. The cholinergic, adrenergic, and glutamatergic pathways are the key points of focus of AD treatment and prevention. In this review, we first present the evidence describing the role of the cholinergic, adrenergic, and glutamatergic systems in AD development. Then, we review the literature on the effects of drugs targeting the cholinergic, adrenergic, and glutamatergic systems in cell cultures, animal models, and human trials of AD. Finally, we detail on how these three systems influence each other.

## 2. Current Etiological Hypothesis of AD Involving the Neurotransmitter System

### 2.1. Cholinergic Hypothesis

The changes in neurotransmitters and their receptors in patients with AD have been well studied. A consistent loss of cholinergic neurons and a considerable decline in choline acetyltransferase activity are the most prominent phenotypes of AD progression. Central cholinergic neurons in the nucleus basalis of Meynert are the primary source of cholinergic innervation to the neocortex. The nucleus basalis of Meynert has been widely studied with respect to AD [5,6,7,8,9], with all pertinent evidence showing that cholinergic neuronal cell degeneration is related to the progressive worsening of memory and cognitive loss in patients with AD. A considerable (29%) overall loss in neuron number was observed in the nucleus basalis of Meynert as well as an even larger loss (61%) in large neurons and a concurrent increase (59%) in small neurons [5].

Both nicotinic acetylcholine receptors (nAChR) and muscarinic ACh receptors have been suggested as drug targets for AD treatment. In the human brain, the heteromeric α4β2 and homomeric α7-nAChR are the major nAChR subtypes [10]. Considerable decreases in α4β2 levels (of up to 50%) have been observed in the brains of patients with AD though an examination of postmortem human brain tissue [11]. Decreases in the α7-nAChR levels observed in the early AD stage are associated with the progression of cognitive deficits [12,13]. However, the results of postmortem protein and mRNA expression studies have led to discrepancies. Although a decrease in α7 protein levels was noted in the cortex and hippocampus [13,14], α7 mRNA levels have been found to be considerably higher in the hippocampus of patients with AD than in that of control patients [15].

The α7-nAChR exhibits high binding affinity to the 42-amino acid Aβ peptide (Aβ(1–42)) [16,17]. Aβ(1–42) can induce cell death in human neuroblastoma cells which overexpress α7-nAChR, and the pretreatment of the α7-nAChR agonists nicotine and epibatidine can protect from Aβ(1–42)-mediated cell toxicity [16]. In one study, chronic nicotine treatment reduced more than 80% of Aβ(1–42)-positive plaques in the brain of a mouse AD model (APPsw) [18]. In addition, α7-nAChR is highly colocalized with Aβ(1–42) within the neurons of AD brains [19]. The crossing of an α7-nAChR-null mutant (α7KO) mouse with a mouse model with AD PDAPP (J9) has been reported to protect from dysfunctions in synaptic integrity and memory behavior [20].

The loss of cholinergic neurons and AChRs in patients with AD makes acetylcholinesterase (AChE) a therapeutic target. Three AChE inhibitors (AChEIs), namely donepezil, galantamine, and rivastigmine, can decelerate acetylcholine breakdown by reducing acetylcholinesterase activity and increasing ACh concentrations at the synapses [21]. The administration of rivastigmine decelerates whole-brain atrophy, hippocampal atrophy, white matter loss, and cognitive decline [22]. Randomized controlled trials on donepezil in patients with AD have indicated that donepezil can also decrease the rate of atrophy in the cortex [23], hippocampus [24], and basal forebrain [25]. Moreover, AChEIs can reduce free radicals and amyloid toxicity as well as cytokine release [26,27,28]. These anti-inflammatory effects demonstrate the positive effects of AChEIs on AD alleviation [29].

### 2.2. Adrenergic Hypothesis

Several studies have shown that the adrenergic system is critical in the CNS. The locus coeruleus (LC), the predominant source of noradrenergic projection neurons, determines the global states of the brain, covering the whole spectrum of brain activation [30], including learning, memory, attention [31,32], sleep–wake cycle regulation [33], active wake and physiological stress [30], as well as aggression regulation. The stimulation of postsynaptic α1-adrenergic receptor (α2AR) with guanfacine can improve working memory performance [34]. Studies have found that LC noradrenergic neurons could be considerably degenerated in the brains of patients with advanced AD [35,36,37,38,39]. A 30% loss in LC neurons was observed during the transition from a state of no cognitive impairment to amnestic mild cognitive impairment (MIC), with an additional 25% loss in LC neurons during AD [40].

Although anatomical and neurotransmitter changes in the adrenergic system have been observed in patients with AD, the expression of adrenergic receptors in postmortem brain tissues reveals an inconsistency. Kalaria showed that α1AR density decreased in the prefrontal cortex [41] but not in the hippocampus, the putamen, or the cerebellum, whereas Szot et al. found that α1AR binding sites increased in layers 1 and 2 of the prefrontal cortex [42]. In the brains of patients with Alzheimer-type dementia, the amount of α1ARs considerably decreased in the hippocampus and the cerebellar hemisphere, whereas that of α2ARs considerably decreased in the nucleus basalis of Meynert [43]. However, in patients with AD, the amount of β1ARs decreased whereas that of β2ARs increased in the cortex, and the amounts of both β1ARs and β2ARs increased in the hippocampus [44].

The distribution of adrenergic receptors in the regions of the brain is correlated with AD pathogenesis. β2ARs are predominant in the human hippocampus, and β1ARs are predominant in the rat hippocampus [45,46]. In addition, βARs play a crucial role in cognitive function. The administration of both selective and nonselective βAR antagonists, including isoproterenol, propranolol, timolol, and sotalol, can impair memory function [47,48,49,50,51]. However, the stimulation of βARs with agonists promotes memory consolidation [50,52,53] and synaptic long-term potentiation (LTP) [54,55,56,57]. In support of the idea that adrenergic neurotransmission participates in memory and leaning, the expression of adrenergic receptors mainly distributes in the dendrites of granule cells and interneurons in dentate gyrus [58,59].

βARs represent a target that might be altered by Aβ oligomers. The direct application of human Aβ oligomers can induce the internalization of transfected human β2ARs in fibroblasts and endogenous β2ARs in rodent prefrontal cortical neurons [60]. Large soluble oligomers of Aβ proteins from human brains with AD are less bioactive than low molecular-weight (approximately 8–70 kDa) Aβ oligomers (which are dissociated from high molecular-weight Aβ oligomers); nevertheless, low molecular-weight Aβ oligomers can decrease the neuron levels of β2ARs, impair hippocampal LTP, and activate microglia in vivo [61]. Furthermore, the binding of soluble Aβ peptides to β2ARs activates G-protein-cAMP-protein kinase A (PKA) signaling and further induces α-amino-3-hydroxy-5-methyl-4-isoxazolepropionic acid receptor (AMPA) receptor hyperactivity [62].

Adrenergic receptor activation restores memory impairment in animal models with AD. In amyloid precursor protein/presenilin 1 (APP/PS1) mice, the β2 adrenergic agonist clenbuterol enhances hippocampal neurogenesis, improves memory deficits, and upregulates dendritic branching and the density of dendritic spines [63]. The administration of the β1AR agonist xamoterol has been found to reduce the social recognition deficit in APP/PS1 mice by increasing nuclear phospho-CREB [64]. These findings suggest that the activation of βAR reduces cognitive deficits in animal models with AD.

### 2.3. Glutamatergic Hypothesis

The glutamatergic pathway, particularly involving *N*-methyl d-aspartate receptor (NMDAR) activation, is the main mediator of synaptic strength and structure, which are related to long-term synaptic plasticity [65]. Ca^2+^ entry via NMDARs and l-type Ca^2+^ channels activate CREB signals in hippocampal pyramidal neurons [66,67]. Because vesicular glutamate transporters (VGLUT1–3) mediate glutamate packing into synaptic vesicles, scientists have used VGLUTs to detect glutamatergic neurons. VGLUT1 mRNA expression is mainly detected in the telencephalic regions, including the cerebral cortex, hippocampus and cerebellum [68,69], whereas VGLUT2 mRNA and proteins are mainly found in the thalamus and lower brainstem regions [69,70]. VGLUT1 and VGLUT2 detection through Western blotting has revealed that VGLUT1 and VGLUT2 are lower in the prefrontal dorsolateral cortices of patients with AD [71]. Many researchers have demonstrated that glutamatergic network dysfunction is associated with AD pathogenesis, which involves a decrease in glutamic acid content and receptor binding in the brain [72,73]. In addition, lowered glutamate uptake levels have been found in the cortex and the hippocampus, indicating glutamatergic synapse loss [74].

Soluble Aβ oligomers stimulate tau phosphorylation [75] and disturb the glutamatergic networks. Aβ42 binds to forebrain synaptosomes, which are associated with postsynaptic density complexes of the NMDA subunits NR1 and NR2B [76]. In cultured rat cortical neurons and entorhinal–hippocampal organotypic slices, soluble Aβ oligomers markedly induced NMDA-dependent inward Ca^2+^ currents and cell apoptosis through the NMDA and AMPA receptors [77]. NMDA and AMPA receptor overactivation leads to mitochondrial dysfunction, including excessive mitochondrial Ca^2+^ and mitochondrial damage. Aβ oligomers can inhibit LTP in hippocampal brain slices, cultured cells, and the cortices of brains with AD [78,79]; this process can be blocked by selective NR2B inhibitors. This observation indicates that soluble Aβ oligomers reduce LTP through the excessive activation of extrasynaptic NMDA receptors containing NR2B.

The overactivation of extrasynaptic NMDARs is linked to neurodegeneration. Memantine, which reduces extrasynaptic NMDAR overactivation [80], has therapeutic effects on moderate to severe AD because it reduces glutamate excitotoxicity [81,82,83]. Compared with other NMDAR antagonists, such as MK-801, the binding affinity of memantine is relatively low. Because of this low binding affinity, memantine strikes a balance between physiological synaptic activity and excessive extrasynaptic activity [84]. Whether NMDAR activation leads to cell survival or cell death depends on the location and strength of NMDAR stimulation. A low amount of NMDAR stimulation activates the synaptic NMDARs, which causes pro-survival signaling, whereas a considerable amount of simulation gradually leads to the stimulation of not only extrasynaptic but also synaptic NMDARs, thus triggering cell death [85]. Therefore, finding a mild NMDAR antagonist has become a research focus in neurodegenerative disease treatment.

NMDAR subunit composition depends on the location. In a mature hippocampus, synaptic NMDARs mainly contain NR2A, whereas extrasynaptic NMDA receptors mainly contain NR2B [86]. The administration of Aβ(1–42) oligomers can induce the nuclear accumulation of Jacob, which is caused by the activation of extrasynaptic NMDARs and correlated with pathological changes in the dendric spines; these effects can be blocked by the NR2B antagonist ifenprodil [87]. NMDARs containing NR2B have high levels of pathological expression with apoptosis at the hippocampus in rat models with AD, indicating the nature of the NR2B–AD relationship [88]. The application of traxoprodil and arcaine, both of which are NR2B antagonists, reverses the Aβ(25–35)-induced reduction in dendritic spine density and morphological changes to the spine [89]. By using an organotypic hippocampal slice from arcAβ transgenic mice combined with overexpressed human tau protein, Tackenberg et al. demonstrated that the blocking of NR2B-containing NMDARs inhibited Aβ-induced tau phosphorylation and cell toxicity because GSK-3β activation was lowered [90]. Although the reduced NR2B activity caused by the receptor antagonists was demonstrated to afford neuroprotection and neuropathic pain alleviation in animal models with Parkinson’s disease [91,92], a clinical trial reported that the effects of traxoprodil, a NR2B antagonist, on traumatic brain injury was nonsignificant [93]. Thus, additional studies investigating NR2B-targeting interventions on dementia are still warranted.

Enhancing NMDAR function through the NMDAR co-agonists d-serine and glycine can offer therapeutic potential. The binding of co-agonists and glutamate are essential for NMDAR activation [94,95,96]. The availability of NMDAR co-agonists varies according to locations. Although both d-serine and glycine originate from glial cells, d-serine is available for synaptic NMDARs and glycine is available for extrasynaptic NMDARs [97]. As stated in Section 2.2, the activation of the synaptic NMDARs plays a key role in neuroprotection, and d-serine is the main co-agonist for NMDARs in the forebrain and the hippocampus [98,99,100]. However, patients with dementia exhibit distinct changes in d-serine levels. Madeira et al. reported that d-serine levels were considerably high in the hippocampus and the parietal cortex in postmortem brains with AD as well as in the cerebrospinal fluid (CSF) of patients with AD [101]. In contrast to the study of Madeira et al., d-serine levels in the CSF of patients with AD did not differ from those in the CSF of the controls [102]. Nagata et al. analyzed the levels of free l-serine and d-serine in the frontal cortex of human brains with and without AD and found no notable difference between the two groups [103]. In a study using blood samples from patients with AD or MIC, patients with poor cognitive function demonstrated high d-serine concentrations [104]. High d-amino acid oxidase (DAAO) levels were also found [105]. The increase in DAAO concentration might have been a secondary response to the high concentration of d-serine, which contributed to late-phase AD because of neurotoxicity.

## 3. Interaction Among Adrenergic, Cholinergic, and Glutamatergic Systems

### 3.1. Link with Adrenergic and Glutamatergic Systems

**Figure 1:**
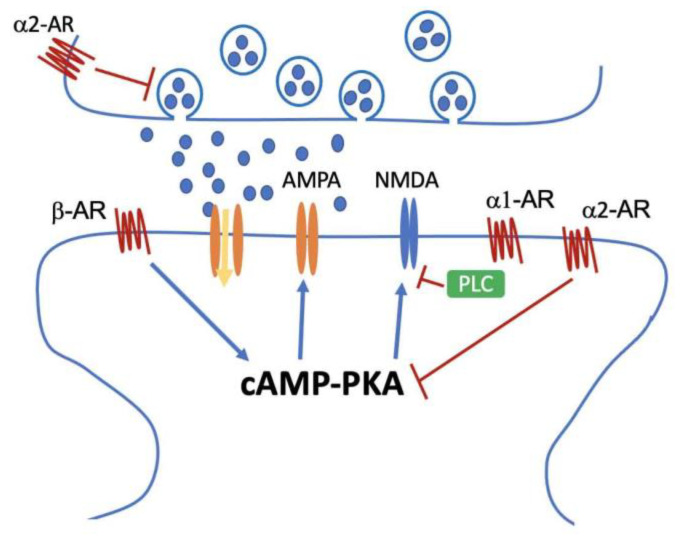
Proposed model of the mechanism of α1, α2, and βAR regulating NMDAR function. βAR enhances NMDAR currents through cAMP-PKA pathways, whereas α2AR reduces NMDAR activity by inhibiting the same pathway or presynaptic glutamate secretion. α1AR, which is a protein-coupled receptor, decreases NMDAR activity through PLC-IP3 signals.

The glutamatergic system plays a key role in controlling cognitive function and working memory in the prefrontal cortex (PFC), but it does not function alone and its connection to other neurotransmitter systems warrants discussion. Because the adrenergic and cholinergic systems are both located on dendritic spines, they are thought to regulate the glutamatergic system and its associated activities [106]. The crossing of *Ear2*^−/−^ mice that had severe LC neuron loss with APP/PS1 mice revealed a decrease in NR2A subunit levels but an increase in NR2B levels [107]. This deficiency in LC neurons causes early memory and learning impairment, suggesting that a reduction in adrenergic stimulation may cause cognitive decline through NMDAR dysfunction. Acute stress activates noradrenaline release in the brain [108]. The binding of noradrenaline to βARs activates PKA and CaMKII, which further phosphorylate GluR1 at S845 and S831. After the systemic administration of epinephrine to increase norepinephrine (NE) levels in the CNS, phosphorylation at Ser845 of GluR1 increases [109]. This phosphorylation can lower the threshold for the incorporation of AMPA receptors containing GluR1 into the synapses during LTP [109], and this synaptic delivery of AMPA receptors is critical to synaptic plasticity [109,110,111,112]. In older rats, impaired LTP is correlated with the misregulation of AMPA receptor trafficking under fear conditioning, and NE administration can reverse LTP impairment by enhancing the GluR1-transporting cell surface [113]. Kobayashi et al. reported that α1AR activation decreased eEPSPs through protein kinase C, whereas the activation of βARs evoked excitatory signals through cAMP-PKA in layer 5 pyramidal neurons [114]. Phenylephrine, the α1AR agonist, decreases miniature EPSC (mEPSC) amplitude. However, the βAR agonist isoproterenol greatly increases the eEPSC amplitude [115]. In addition to AMPA receptors, the application of NE and NE transporter inhibitors reduces the amplitude of EPSC mediated by NMDA receptors in the PFC pyramidal neurons [116]. A possible mechanism is that G-protein-coupled α1AR decreases NMDAR currents via the PLC-IP3 pathway, which is regulated by regulators of G-protein signaling 2. Activation of α2AR reduces PKA–ERK signaling and furthers NMDAR transports, leading to the downregulation of NMDAR currents [116]. Moreover, presynaptic α2AR inhibits glutamate transmission in the ventral tegmental area [117]. Because the activation of α1, α2, and βARs results in various effects for AMPA and NMDA receptors, it reveals that the effects of NE on glutamatergic receptors depend on each individual cell and receptor [118,119,120]. Figure 1 illustrates the mechanism of α1, α2, and βAR regulating NMDAR function.

### 3.2. Link to Cholinergic and Glutamatergic Systems

The most frequently discussed topic in studies on connections with the cholinergic and glutamatergic systems is that of the dorsolateral PFC (dlPFC). Nicotinic α7-nAChR is enriched in the glutamate network synapses in the dlPFC and is required for NMDA action, indicating that α7-nAChR and NMDARs work together in dlPFC circuits [121]. However, the action of acetylcholine on NMDA may be region dependent. In CA1 neurons of the hippocampus, ACh potentiates NMDA receptors through muscarinic receptors [122] and possibly through the inositol 1,4,5-trisphosphate pathway in the hippocampus [123]. Exposure of the cholinergic receptor agonist carbachol in the hippocampus can induce LTP, which is dependent on NMDA receptor activation [124]. By contrast, NMDAR-mediated currents can be directly inhibited through the application of acetylcholine in the cortical brain slice, and this inhibition is independent of G-protein and voltage [125]. The administration of the AChE inhibitor donepezil can significantly reduce the surface expression of NR1, the core subunit of NMDAR, and glutamate-induced toxicity. α7-nAChR blockage by methyllycaconitine reduces the donepezil-induced attenuation of glutamate-mediated Ca^2+^ entry [126]. Physostigmine, another AChE inhibitor, reduces NMDAR-mediated excitatory postsynaptic currents through Ca^2+^ and the ERK pathway [127]. nAChR activation can cause increases in neurotransmission and selective increases in the amplitudes of AMPA receptor (AMPAR)-mediated currents in layer 1 neurons in the PFC [128]. Nicotine increases intracellular calcium levels through α7-nAChR and modulates the phosphorylation of the GluR1 AMPAR subunit, which leads to an increase in AMPAR current. Nicotine-treated astrocytes exhibits high AMPAR levels in the hippocampal slices. This activation of α7-nAChR on astrocytes lead to AMPAR recruitment to the postsynaptic sites on the neuron surface [129].

The cholinergic and glutamatergic systems may be connected by d-serine. The binding of d-serine or glycine at the glycine modulatory site is essential to activate NMDARs [96]. Astrocytes produce l-serine from glucose, and l-serine is then transported to neurons and converted to d-serine by serine racemase [130,131]. Thus, serine racemase modulation plays a key role in NMDAR activity. In PC-12 phaeochromocytoma cells and 1321N1 astrocytoma cells, incubation with α7-nAChR antagonists decreases the expression of serine racemase [132]. The deletion of the α7-nAChR gene in mice can cause the loss of d-serine and NMDARs, which leads to glutamatergic synaptic deficiency in the cortex [133]. The stimulation of the nucleus basalis of Meynert, the major source of cholinergic innervation to the cortex, considerably increases d-serine levels in wild type mice compared with those in inositol-1,4,5-trisphosphate receptor type 2 knockout mice [134]. Moreover, this elevation in d-serine levels is involved in ACh-modulated and NMDAR-dependent synaptic plasticity.

## 4. Potential Therapeutic Targets of NMDAR Enhancers

Because the adrenergic, cholinergic, and glutamatergic systems influence each other and because synapses containing NMDARs are the main target of cognition dysfunction, most of the therapeutic strategies have focused on modulating glutamate neurotransmission [135]. Although glutamate excitotoxicity through NMDARs is the primary cause of acute neuronal injuries, the direct administration of NMDAR antagonists, such as ketamine and MK-801, may increase dopamine release and schizophrenia-like behavior [136,137,138,139,140,141,142]. Thus, the use of NMDAR co-agonists to alter NMDA neurotransmission and improve cognition has become an alternative method for modulating NMDARs. Several types of NMDA-enhancing agents targeting co-agonist sites have been identified. Glycine and d-serine are two co-agonists that bind to segments S1 and S2 on the GluN1 and GluN3 subunits of NDMARs [143]. The co-agonist site can be activated by full agonists, including glycine and d-serine. The administration of d-serine as a supplement in water can prevent immune-induced cognitive deficits in adult offspring after maternal exposure to poly(I:C) [144]. Acute oral administration of d-serine in older adults can improve spatial learning and problem-solving abilities [145]. Patients with schizophrenia who received d-serine treatment to enhance NMDARs were noted to exhibit notable improvements to cognitive impairment as well as to positive and negative symptoms [146,147]. However, a multicenter add-on randomized controlled trial of low-dose d-serine treatment in patients with schizophrenia did not reveal any notable differences between the d-serine treatment group and the placebo group [148]. Therefore, longer-term, higher-dose d-serine treatment regimens in patients with cognitive impairments should be investigated in future studies.

The second method for enhancing co-agonist sites is through partial agonists, such as d-cycloserine. d-cycloserine can facilitate NMDAR–ionophore complex activation and enhance cognition in patients with AD [149]. d-cycloserine can reverse scopolamine-induced acquisition performance impairment in rats [150]. In a study on the effects in humans, Jones et al. pretreated healthy patients with the anticholinergic drug scopolamine as a model for memory impairment associated with AD. The authors found that low doses of d-cycloserine had a positive effect on memory [151]. The short-term treatment with 100 mg/day d-cycloserine is associated with notable improvements in cognitive function [152,153]. However, several studies have indicated that d-cycloserine does not benefit patients with AD [154,155,156]. The results of two large and two small randomized controlled trials suggested that d-cycloserine does not improve cognitive impairments in patients with AD [157]. Another reason for the limited use of d-cycloserine is the side effects, include hyperexcitability, dizziness, depression, anxiety, confusion, memory loss, and lethargy, which were found in using high dose of d-cycloserine [158].

Another method is to alter d-serine and glycine levels by modulating their generators and metabolizers. Increasing synaptic glycine by using glycine transporter-1 inhibitors ASP2535 or TASP0315003 can reduce cognitive deficits in rodent models with schizophrenia and AD [159,160]. A recent study evaluated the efficacy and safety of orally administered BI 425809, a selective GlyT1 inhibitor, for patients with AD exhibiting cognitive deficits. However, no notable changes were observed after 12 weeks of treatment [161]. Another method for enhancing NMDAR levels through increasing d-serine levels is to reduce d-serine degradation through inhibiting DAAO, a primary mediator of d-serine metabolism in the brain [162,163]. The d-serine levels in mice increased after administration of sodium benzoate and PGM030756, both of which are DAAO inhibitors [164]. Several studies have evaluated the clinical effects of DAAO inhibitors on patients with schizophrenia [162,165,166] and dementia [167,168,169], and their results have suggested that sodium benzoate has potential for delaying AD progression. Sodium benzoate can reduce the activation of p21rac, oxidative stress, and neuronal apoptosis in the hippocampus and can protect against the degeneration of spatial learning and memory capabilities in 5XFAD Tg mice [170]. A randomized, double-blind, placebo-controlled trial on the use of sodium benzoate demonstrated that sodium benzoate can improve cognitive and overall function in patients with MIC or mild AD [167]. A 6-week sodium benzoate treatment in patients with behavioral and psychological symptoms of dementia did not yield any notable improvements [168]. Combined with a precision medicine approach, a 6-week treatment of sodium benzoate demonstrated potential to improve cognitive function in some patients with behavioral and psychological symptoms of dementia [169]. Through measures of working memory, verbal learning, and regional homogeneity maps, sodium benzoate has been demonstrated to modify brain activity and cognition in patients with MCI [171]. Among these clinical studies, no adverse effects were observed in line with small doses of sodium benzoate have little or no side effect on animals [172]. Taken together, sodium benzoate shows greater therapeutic potential in the clinical treatment of AD than other NMDAR enhancers.

## 5. Conclusions

The cholinergic, norepinephrine, and glutamatergic networks and their interactions are involved in cognitive dysfunction associated with AD, with the glutamatergic system playing an essential role in the regulation of synaptic plasticity and cognition. The modulation of the glutamatergic system and NMDAR-based enhancement therapy has been effective in some clinical trials. However, clinical trials have not yet determined potentiators for NMDAR activation in AD cases. The modulation of NMDAR co-agonist levels also benefits patients with other cognitive disorders, including schizophrenia. However, further research on the efficacy and safety of this treatment in both early and late AD stages is warranted.
